# A novel application of RNase H2-dependent quantitative PCR for detection and quantification of *Grosmannia clavigera*, a mountain pine beetle fungal symbiont, in environmental samples

**DOI:** 10.1093/treephys/tpx147

**Published:** 2018-01-10

**Authors:** Chandra H McAllister, Colleen E Fortier, Kate R St Onge, Bianca M Sacchi, Meaghan J Nawrot, Troy Locke, Janice E K Cooke

**Affiliations:** Department of Biological Sciences, University of Alberta, Edmonton, AB, Canada T6G 2E9

**Keywords:** bark beetles, blue-stain fungi, defense responses, forest biotechnology, forest pathology, pine

## Abstract

Mountain pine beetle (*Dendroctonus ponderosae* Hopkins; MPB) is an economically and ecologically important pest of pine species in western North America. Mountain pine beetles form complex multipartite relationships with microbial partners, including the ophiostomoid fungi *Grosmannia clavigera* (Robinson-Jeffrey and Davidson) Zipfel, de Beer and Wingfield, *Ophiostoma montium* (Rumbold) von Arx, *Grosmannia aurea* (Robinson-Jeffrey and Davidson) Zipfel, de Beer and Wingfield, *Leptographium longiclavatum* (Lee, Kim, and Breuil) *and Leptographium terebrantis* (Barras and Perry). These fungi are vectored by MPB to new pine hosts, where the fungi overcome host defenses to grow into the sapwood. A tree’s relative susceptibility to these fungi is conventionally assessed by measuring lesions that develop in response to fungal inoculation. However, these lesions represent a symptom of infection, representing both fungal growth and tree defense capacity. In order to more objectively assess fungal virulence and host tree susceptibility in studies of host–pathogen interactions, a reliable, consistent, sensitive method is required to accurately identify and quantify MPB-associated fungal symbionts in planta. We have adapted RNase H2-dependent PCR, a technique originally designed for rare allele discrimination, to develop a novel RNase H2-dependent quantitative PCR (rh-qPCR) assay that shows greater specificity and sensitivity than previously published PCR-based methods to quantify MPB fungal symbionts in pine xylem and MPB whole beetles. Two sets of assay probes were designed: one that amplifies a broad range of ophiostomoid species, and a second that amplifies *G. clavigera* but not other MPB-associated ophiostomoid species. Using these primers to quantify *G. clavigera* in pine stems, we provide evidence that lesion length does not accurately reflect the extent of fungal colonization along the stem nor the quantity of fungal growth within this colonized portion of stem. The sensitivity, specificity, reproducibility, cost effectiveness and high-throughput potential of the rh-qPCR assay makes the technology suitable for identification and quantification of a wide array of pathogenic and beneficial microbes that form associations with plants and other organisms, even when the microbial partner is present in low abundance.

## Introduction

Mountain pine beetle (*Dendroctonus ponderosae*; MPB) is a bark beetle that is native to western North America (Safranyik et al. 2010). These beetles form complex multipartite symbioses with a variety of microorganisms, including bacteria (Winder et al. 2010) and fungi (Roe et al. 2010), which allows them to effectively attack and feed on a variety of pine species, including lodgepole pine (*Pinus contorta* Dougl. ex Loud. var. *latifolia*) (Safranyik et al. 2010), jack pine (*Pinus banksiana* Lamb.) and their hybrids (Cullingham et al. 2011). Several species of MPB fungal associates are wood-colonizing filamentous fungi belonging to family Ophiostomataceae within the Ascomycota, and are commonly referred to as the ophiostomoid or blue-stain fungi (Alamouti et al. 2011, Six and Wingfield 2011). These ophiostomoid fungi can be found on the surface of the exoskeleton and in the gut of MPB at different life stages, including larvae and adults; adult MPB also carry spores of fungal associate species in specialized invaginations of the exoskeleton called mycangia (Six 2003). The most commonly encountered ophiostomoid MBP associates are: *Grosmannia clavigera* (Robinson-Jeffrey and Davidson) Zipfel, de Beer and Wingfield; *Leptographium longiclavatum* (Lee, Kim, and Breuil); and *Ophiostoma montium* (Rumbold) von Arx (Lee et al. 2005, 2006, Roe et al. 2010, 2011*a*). Other species of ophiostomoid fungi such as *Ceratocystiopsis* sp. 1 (Lee et al. 2006), *Grosmannia aurea* (Robinson-Jeffrey and Davidson) Zipfel, de Beer and Wingfield, and *Leptographium terebrantis* (Barras and Perry) have been observed to associate with MBP, albeit at a lower rate; these fungal species are also associated with other bark beetle species (Six 2003, Bleiker and Six 2007, Lieutier et al. 2009, Roe et al. 2010). The relationship between MPB and these ophiostomoid fungal species is considered to be mutualistic, whereby the fungi are vectored to new host trees by MPB while the fungi contribute to capacity of MPB to overcome the host tree’s defense systems and also serve as a nutrient source to colonizing beetles and their offspring (Six and Wingfield 2011). Although it is clear that at least some of these ophiostomoid fungal associates are phytopathogenic (Solheim and Krokene 1998, Rice et al. 2007, Plattner et al. 2008), the extent to which ophiostomoid fungi contribute to the beetle’s ability to overcome tree defenses remains contentious (Six and Wingfield 2011).

Several studies indicate that there is intra- and interspecific variation in the virulence of ophiostomoid fungi associated with MPB (Solheim and Krokene 1998, Rice et al. 2007, Plattner et al. 2008, Rice and Langor 2008, Bleiker and Six 2009*a*, 2009*b*) with *G. clavigera* and *L. longiclavatum* being considered more virulent than *O. montium* (Rice et al. 2007, Rice and Langor 2008). Furthermore, the relative proportion of species within the fungal community associated with larval or adult MPB varies considerably, with the community composition affected by abiotic factors such as temperature as well as biotic factors such as competitive interactions between fungal species and other species within the MPB microbiome (Rice et al. 2007, Roe et al. 2011*a*). It has been hypothesized that fungal community composition may influence MPB success in attacking and colonizing its pine host (Roe et al. 2011*a*). Classically, fungal virulence has been assessed by comparing reaction zones that arise following inoculation (Reid et al. 1967, Lee et al. 2006, Rice and Langor 2008). These reaction zones—referred to as lesions—manifest in both xylem and phloem, and comprise cells that accumulate considerable quantities of defensive chemicals such as terpenoid and phenolic compounds (Raffa 1991, Franceschi et al. 2005). Lesion length following fungal inoculation is also used as a relative measure of a tree’s defensive capacity against these fungi (Masuya et al. 2003, Rice et al. 2007, Arango-Velez et al. 2014). Indeed, inoculation with MPB fungal associates is often used as a proxy for MPB attack (Wong and Berryman 1977, Rice et al. 2007).

PCR-based techniques are routinely used to quantify fungal growth of foliar pathogens as a means to compare resistance levels of different host genetic backgrounds or virulence of different pathovars in crop plants (Reischer et al. 2004, Boutigny et al. 2012), woody plants (Boyle et al. 2005) and other angiosperms (Hacquard et al. 2011, Sanzani et al. 2014). PCR has been used more recently to identify and quantify fungal colonization in wood and/or bark samples from forest trees (Song et al. 2013, Strid et al. 2014, Matsiakh et al. 2016). PCR-based approaches have also been used to detect fungi associated with bark beetles (Strid et al. 2014, Floren et al. 2015). The more novel loop-mediated isothermal amplification (LAMP) technology has been used to detect the ophiostomoid fungus *Ophiostoma clavatum* Math.-Käärik in DNA of *Ips acuminatus* (Gyll.) bark beetles (Villari et al. 2013). Recently, next generation sequencing of the rDNA ITS2 region was used to identify the fungal community metagenome of a bark beetle *Orthotomicus erosus* (Wollaston) and an ambrosia beetle *Xyleborinus saxesenii* (Ratzeburg) (Malacrinò et al. 2017). Next generation sequencing has also been exploited to identify the bacterial community metagenome of MPB (Adams et al. 2013), but to our knowledge has not been used to characterize the fungal community.

Most of the abovementioned molecular analyses of forest tree- or bark beetle-associated fungi have focused on identification rather than quantification of the fungal associate species. Similarly, the PCR-based techniques that have been developed to detect MPB-associated ophiostomoid fungal species have focused on discrimination between different ophiostomoid species (Khadempour et al. 2010, Tsui et al. 2010), and were not explicitly designed to quantify MPB fungal associates in host tree or MPB tissues. Only one study to date has used qPCR to assess relative abundance of different fungal taxa in woody tissue, although the markers could not distinguish *G. clavigera* from *L. longiclavatum* (Khadempour et al. 2012). Other methods, such as microscopy (Ballard et al. 1982, 1984, Woo et al. 2005) or regrowth of fungi from colonized wood or MPB tissues (Roe et al. 2010, 2011*a*, 2011*b*) are time-intensive, difficult to scale up, and qualitative rather than quantitative. Furthermore, culturing of MPB fungal symbionts can lead to biased estimates of community representation, since faster growing isolates are more likely to be retrieved than slower growing isolates.

The objective of this study was to design an improved PCR-based assay to accurately quantify MPB-associated ophiostomoid species from environmental samples such as pine or MPB tissues, focusing on *G. clavigera* as the most prevalent and pathogenic fungal associate of MPB (Rice et al. 2007, Plattner et al. 2008, Roe et al. 2011*b*). Here we present a sensitive and specific quantitative PCR (qPCR) assay that utilizes RNase H2-dependent technology (Dobosy et al. 2011) to quantify ophiostomoid fungi in complex samples such as pine and MPB. RNase H2-dependent PCR, which was originally designed to detect rare alleles in single nucleotide polymorphism (SNP) variant discrimination, uses a modified primer design that incorporates a single RNA base at or near the 3′-end. This RNA residue effectively blocks extension by the DNA polymerase until it is removed through the action of a thermostable RNase H2. The efficacy of the RNase H2 cleavage reaction is substantially reduced when mismatches are present at or near the RNA:DNA site, thus decreasing primer dimers and off-target amplification. We developed a generic RNase-H2 quantitative PCR (rh-qPCR) assay for detection and quantification of multiple ophiostomoid species from the *Grosmannia* clade (sensu Zipfel et al. 2006), and a specific rh-qPCR assay for *G. clavigera* as the most prevalent and pathogenic fungal associate of MPB. We compared the performance of these rh-qPCR assays to histochemical methods commonly used to detect fungal pathogens in plant tissues, and also to a conventional qPCR assay using primers modified from Khadempour et al. (2010). We then used these two rh-qPCR assays to quantify ophiostomoid fungi and specifically *G. clavigera* in xylem tissue from lesions of pine seedlings and mature trees, as well as in whole MPB adult beetles, evaluating the utility of the rh-qPCR assays for molecular investigations of plant–pathogen interactions and microbiome communities. We discuss the applicability of the methodology that we have developed for detection and quantification within other pathosystems, as well as for diverse microbes within a broad range of complex environmental samples.

## Materials and methods

### Biological materials

Several sets of experimental materials were generated for evaluation of the rh-qPCR assay primers.

#### Ophiostomoid species panel

Multiple isolates of the *D. ponderosae* ophiostomoid fungal associates *G. clavigera*, *G. aurea*, *O. montium, L. longiclavatum* and *L. terebrantis* were obtained through the UAMH, formerly known as the University of Alberta Microfungus Collection and Herbarium (Edmonton, AB, Canada), now called the UAMH Centre for Global Microfungal Biodiversity (University of Toronto, Toronto, ON, Canada). Identifying information for these isolates is outlined in Table 1. Each of these fungal strains was previously collected, isolated, identified and vouchered by Roe et al. (2010, 2011*a*, 2011*b*). Each fungal isolate represents a culture derived through single spore isolation, and the species for each isolate was assigned through multilocus sequencing of *actin*, *elongation factor 1 alpha* (*EF1a*), *beta-tubulin*, an anonymous nuclear locus and *ITS2* (partial 5.8S + internal transcribed spacer region 2 + partial 28S) as described in Roe et al. (2010, 2011*b*). NCBI accession numbers for the sequences used in this study are listed in Table S1 available as Supplementary Data at *Tree Physiology* Online. Cultures of other ophiostomoid fungi known to associate with different species of bark beetles—*Leptographium abietinum* (Peck) Wingfield, *Leptographium procerum* (Kendrick) Wingfield and *Ophiostoma minus* (Hedgcock) Sydow and Sydow—were also obtained from the UAMH (Table 1). *Ophiostoma ips* (Rumbold) Nannfeldt (Table 1) was obtained from the Natural Resources Canada, Canadian Forest Service, Northern Forestry Centre Mycological Herbarium (NoF), with ID NoF 1205, Specimen no. CFB 21,605. These non-MPB fungal associate cultures were not single spore isolates.

**Table 1.** Fungal isolates used in this study. Each fungal isolate with a unique ID from Roe et al. (2010, 2011*b*) represents a single spore culture. Species identities of these 15 isolates were determined by multilocus sequencing of *β-tubulin*, *EF1-α*, *actin* and *ITS2* as described in Roe et al. (2010, 2011*b*). All cultures were obtained from the UAMH except *O. ips*, which was obtained from the Northern Forestry Centre Mycological Herbarium.

UAMH ID	Unique ID from Roe et al. (2010, 2011*b*)	Species	Isolate origin	Sampling coordinates
11,139	M001-02-03-05-UC17DL22 SS124	*G. clavigera*	Willmore-Kakwa, Alberta	53.8233, −119.6641
11,143	M002-06-01-03-UM01G12 SS163	*G. clavigera*	Canmore, Alberta	50.9333, −115.3356
11,147	M002-12-03-03-UC10G11 SS496	*G. clavigera*	Sparwood, British Columbia	49.8895, −114.9146
10,965	M001-03-03-06-UC03DL06 SS98	*G. aurea*	Fox Creek, Alberta	54.4812, −116.6348
10,969	M002-03-01-12-UC16G21 SS471	*G. aurea*	Fox Creek, Alberta	54.7964, −116.6522
10,970	M002-06-01-08-UL05DL39 SS12	*G. aurea*	Canmore, Alberta	50.9710, −115.3389
11,037	M001-02-01-01-UM02DL02 SS196	*O. montium*	Willmore-Kakwa, Alberta	53.7129, −119.7432
11,038	M001-02-01-07-UM34G38 SS186	*O. montium*	Willmore-Kakwa, Alberta	53.7016, −119.7537
11,043	M001-13-03-03-UM04G04 SS315	*O. montium*	Fairview, Alberta	56.1826, −118.6345
11,014	M002-03-01-17-UC47DA13G63 SS422	*L. longiclavatum*	Fox Creek, Alberta	54.7299, −116.5016
11,017	M002-06-05-14-UL03DL74 SS335	*L. longiclavatum*	Canmore, Alberta	51.1093, −115.3431
11,019	M002-11-03-04-UL02G20 SS504	*L. longiclavatum*	Golden, British Columbia	51.4972, −117.0035
11,001	M001-07-01-03-UC23DL10 SS470	*L. terebrantis*	Crowsnest Pass, Alberta	49.6293, −114.6956
11,006	M002-07-02-01-UC08DL08 SS474	*L. terebrantis*	Crowsnest Pass, Alberta	49.9856, −114.4947
11,007	M002-16-01-05-UC38DL41 SS483	*L. terebrantis*	Yoho National Park, British Columbia	50.9134, −115.9620
4800		*L. abietinum*	Revelstoke, British Columbia	
4917		*O. minus*	Waterton, Alberta	
9724		*L. procerum*	Taupo, Kaingaroa State Forest, New Zealand	
		*O. ips*	Invermere, British Columbia	

Freeze-dried fungal samples obtained from the UAMH were reconstituted in sterile water and cultured on potato dextrose agar at room temperature and ambient lighting for 2 weeks. All fungal cultures were maintained on malt extract agar (MEA) at room temperature and ambient lighting. Fungi to be harvested and used for DNA extractions were grown on MEA overlaid with autoclavable cellophane membrane (BioRad, Hercules, CA, USA), with harvesting conducted after 2 weeks of growth. Harvested mycelia were flash frozen in liquid nitrogen and stored at −80 °C until extraction.

#### *G. clavigera-*inoculated mature lodgepole pine

A field study was performed between July and August 2016, using a natural stand of even-aged mature lodgepole pine trees (54°27′N, 118°37′W), located ~91 km south of Grande Prairie, AB, Canada.

Thirty healthy lodgepole pine trees with no signs of recent damage or disease were randomly assigned to three treatments groups: control uninoculated, wounded but not inoculated (mock) and inoculated. Tree diameters at breast height were 30.7 ± 3.4 cm (±SD, *n* = 30). Trees were inoculated at 1.3 m using a 1.25 cm round leather punch (Tandy Leather, Edmonton, AB, Canada) to puncture the bark at multiple points of the main stem of each tree, and an agar-mycelium plug of *G. clavigera* isolate M002-12-03-03-UC10G11 SS496 (described by Roe et al. 2010, 2011*b*) grown on MEA was inserted into each hole. Each tree received six to eight inoculations spanning approximately half the tree’s circumference. Inoculation sites were wrapped in shrink wrap for 2 weeks. Mock trees were wounded as above, but sterile MEA was inserted into the hole before wrapping. Control trees were neither wounded nor inoculated.

Five weeks post-inoculation, the bark was cut and peeled away from one lesion on each of six trees randomly selected from each treatment group, and xylem samples were collected from one lesion per tree at 2.5 cm intervals from the point of inoculation down to the lower tip of the lesion, using the 1.25 cm round leather punch. For mock trees, the wound site and one spot each above and below the wound was sampled. For control trees, samples were taken at equivalent heights to the wound trees (1.3 m, and one each above and below). Samples were flash frozen in liquid nitrogen and kept on dry ice for transport. Samples were stored at −80 °C prior to extraction.

#### *G. clavigera*-inoculated lodgepole, jack and hybrid pine seedlings

A full-factorial experiment was conducted with three factor levels: pine species (lodgepole, jack and hybrid pine), water treatment (well-watered and water deficit) and days post-inoculation (dpi) with *G. clavigera*. Pine seedlings in their second year of growth were potted in 1.2 l pots with 2:1 peat moss and vermiculite, and the experiment was carried out in a controlled environment growth room at 22 °C (day/night) with 16 h light/8 h dark photoperiod, ~ 250 μmol photosynthetically active radiation and 70–80% relative humidity. For the first 6 weeks, seedlings were watered to soil capacity twice weekly. Once a week for 2 weeks, seedlings were fertilized with 0.5 g l^−1^ 15–30–15 N–P–K (Plant Products Company Ltd, Brampton, ON, Canada), then subsequently on a weekly basis with 0.5 g l^−1^ 20–20–20 N–P–K. Six weeks after potting, the water treatment was initiated. Trees in the well-watered group were watered and fertilized as above, while trees in the water-deficit group were watered twice weekly with 30–50 ml of water, and once weekly with 40 ml of 0.5 g l^−1^ 20–20–20 N–P–K fertilizer solution. The soil relative water content in the water-deficit treatment was maintained at 10–15%, as determined by time-domain reflectometry using methods described in Arango-Velez et al. (2011). The physiological status of the well-watered vs water-deficit treatments was monitored by measuring light-saturated net photosynthesis rate with a portable infrared gas analyzer (Li 6400 photosynthesis system, LI-COR, Lincoln, NE, USA). Four weeks after the initiation of the water-deficit treatment, which was sufficient to affect photosynthesis, plants were inoculated with the same *G. clavigera* isolate M002-12-03-03-UC10G11 SS496 by using a blunt-tipped needle to puncture the bark of the main stem in two locations per tree: 1 μl of spore suspension (~ 2000 spores μl^−1^) was applied into the wound, and inoculation sites were wrapped in Parafilm. At 7, 15 and 29 dpi, bark from six seedlings per treatment combination was peeled and lesions lengths on the xylem surface were measured. An 8–10 cm segment of xylem containing both lesions was flash frozen and stored at −80 °C prior to extraction. Stem tissue from seedlings in their second year of growth was collected at 42 dpi from a similar experiment for histochemical analyses; this experiment is described in detail in Arango-Velez et al. (2016).

#### Mountain pine beetle panel

Twelve adult beetles were collected from three independent MBP-infected trees within a stand located at 52.849°, −118.572° within Mount Robson Provincial Park, British Columbia, Canada, in November 2015, with three to five beetles collected from each tree. Beetles were stored at −20 °C prior to extraction.

### Histology

Histochemical analyses to detect fungal hyphae in *G. clavigera*-inoculated pine stems were carried out using three staining procedures commonly used to detect fungal mycelia in fresh or fixed plant tissues: periodic acid–Schiff (PAS), Toluidine Blue O (TBO) and Calcofluor White. Five millimeter sections collected within the lesion—one at the wound site, two above and two below—were placed directly into fixative (2% (v/v) glutaraldehyde, 1% (w/v) caffeine, 0.1 M sodium phosphate buffer (pH 7.2)), fixed under vacuum for 1 week, then washed with 0.1 M sodium phosphate buffer, dehydrated in an ethanol series, infiltrated and embedded in JB-4 Plus (Polysciences, Hatfield, PA, USA) at 52 °C (Arango-Velez et al. 2016). Three micrometer sections were cut with glass knives for histochemical staining. Periodic acid–Schiff staining was carried out according to Arango-Velez et al. (2016). Sections stained using TBO were immersed in 0.1% (w/v) TBO in 0.5 M acetate buffer, pH 4.4, for 1 min 15 s then air-dried. For staining of tissues with Calcofluor White, one drop of Calcofluor White Stain (Millipore Sigma, Oakville, ON, Canada) and one drop of 10% KOH were added to sample slides; slides were incubated 1 min before observation with fluorescence microscopy. Light microscopy was carried out with a bright field light microscope (Zeiss Axio Scope A1, Oberkochen, Germany, Optronics camera picture frame 2.3, Tulsa, OK, USA). Fluorescence microscopy (Leica model no. DMRXA, Wetzlar, Germany) was carried out in epifluorescence mode with an A4 filter cube (excitation wavelength 360 nm, dichromatic mirror wavelength 400 nm, suppression wavelength 470 nm) and captured using a QIClick camera (Q-Capture Pro7 software).

### DNA extraction

Frozen pine, beetle and fungal samples were ground using a Mixer Mill MM 301 (Retsch, Haan, Germany) or a Geno/Grinder 2010 (Spex SamplePrep, Metuchen, NJ, USA). DNA was extracted using a hexadecyltrimethylammonium bromide (CTAB) protocol (Chang et al. 1993) modified according to Roe et al. (2010). Single beetles were treated as extraction units whereas pine samples were standardized to ~150 mg of finely ground pine tissue per DNA extraction. Samples were analyzed for nucleic acid concentration and purity using an Infinite M200 PRO NanoQuant (Tecan, Männedorf, Switzerland) prior to qPCR.

### rh-qPCR: primer design, PCR conditions and standard curves

Primers were designed using RNase-H2 Generation 1 technology (Integrated DNA Technologies, IDT, Coralville, IA, USA) (Dobosy et al. 2011). These specialized primers contain an RNA base and a C3 spacer which blocks the 3′-end of the primer. Addition of RNase-H2 enzyme to the PCR results in cutting of the primer at the RNA base when specifically paired to a DNA template, releasing the C3 spacer and allowing for elongation from the 3′-end of the primer (Dobosy et al. 2011). Candidate targets for rh-qPCR primer design were chosen from the five loci used by Roe et al. (2010, 2011*b*) for multilocus sequence typing of *Grosmannia* clade ophiostomoid species (sensu Zipfel et al. 2006) isolated from MPB or galleries collected from 45 stands in 12 different landscapes in Alberta and British Columbia, Canada. In these studies, *actin*, *elongation factor 1 alpha*, *β-tubulin*, an anonymous nuclear locus and *ITS2* (comprising partial 5.8S + internal transcribed spacer + partial 28S) had been Sanger sequenced for more than 500 single spore isolates randomly selected from 5063 isolated, morphotyped strains that included *G. clavigera*, *L. longiclavatum*, *O. montium*, *L. terebrantis* and *G. aurea*. This relatively deep set of sequences across multiple genotypes of these key MPB fungal associate species revealed portions of these locus sequences shared by or differentiating these species, while comparison of these sequences to pine or MPB sequences enabled design of primers to minimize possible off-target amplification. Based on the Roe et al. (2010, 2011*b*) data, primers were designed to amplify a portion of the 28S region that was common to these ophiostomoid species. Primers targeting *β-tubulin* were designed to specifically discriminate *G. clavigera* from other ophiostomoid fungal species associated with *D. ponderosae*. These are referred to as the 28S and Gc primers, respectively. A third set of conventional qPCR primers against the same *G. clavigera*-specific *peroxisomal-coenzyme A synthetase* (*PCAS*; Genbank HQ633743) target as used by Khadempour et al. (2010) were also designed. These primers were slightly modified to reduce the high degree of primer dimers that were observed with the published primers, and are referred to as the PCAS primers. Primer details are given in Table 2.

**Table 2.** Primers used for quantification of ophiostomoid fungi in pine and mountain pine beetle tissues. For RNase-H2 primers, 28S and Gc, the RNA base in each primer is underlined, while the C3 spacer at the 3′-end of each primer is denoted by ‘x’.

Primer set	Target	RNase-H2 primers		Specificity	Amplification efficiency
		Forward	Reverse		
28S	28S rDNA	5′-gcctagcctctgtgaagctccttcc_x_-3′	5′- ccctcttttcaaagtgcttttcatcttc_x_- 3′	All species tested in this study	96.141 (*R*^2^ = 0.997, slope = −3.418)
Gc	*β-tubulin*	5′-gtaggtttccggtttcggaagagtctattt_x_-3′	5′-cggcgcggggcacatactc_x_-3′	*G. clavigera*	123.459 (*R*^2^ = 0.98, slope = −2.864)
PCAS	*Peroxisomal-coenzyme A synthetase*	5′-gacgggcctgcttagtaaat-3′	5′-gcgagagagaggagagagag- 3′	*G. clavigera*	106.289 (*R*^2^ = 0.998, slope = −3.18)

qPCR and rh-qPCR was carried out in 10 μl reactions using a 7900HT Fast Real-Time System (Applied Biosystems, Foster City, CA, USA). Three technical replicates were conducted for each biological sample. Reaction mixtures contained 2 μl DNA template (~10–25 ng), 5 μl of SYBR Green master mix (Tris (pH 8.3), KCl, MgCl_2_, glycerol, Tween-20, DMSO, dNTPs, ROX, SYBR Green (Molecular Probes, Waltham, MA, USA), and an antibody inhibited Taq polymerase), 400 nM of each primer and 2.6 mU RNase-H2 (IDT). Primers for multiple targets were not multiplexed. Reaction conditions were as follows: 95 °C for 2 min, 30–50 cycles at 95 °C for 15 s then 60 °C for 1 min. Following the final 1 min at 60 °C, a dissociation step (95 °C for 15 s, 60 °C for 1 min, 95 °C for 15 s) to detect product melting range analysis was performed. Species discrimination of fungal samples from cultures were analyzed for a total of 30 reaction cycles, as this was sufficient to generate considerable signal, whereas fungal detection in beetle and pine samples were monitored for a total of 40 and 50 reaction cycles, respectively. Fungal detection in pine samples was carried out using additional reaction cycles to maximize the limit of detection of low-abundance fungal colonization. Uninoculated lodgepole pine DNA was used as a negative control, while custom-synthesized double-stranded linear DNA (gBlock; IDT) containing the conserved partial 28S rDNA region for the ophiostomoid fungi was used as a positive control for the 28S primers (see Figure S1 available as Supplementary Data at *Tree Physiology* Online).

Standard curves were produced using DNA either from fungal cultures of *G. clavigera* or the 28S gBlock. All standard curves ranged from ~0.001 to −30 ng fungal DNA per reaction. Three technical replicates were conducted for each standard curve point. Curves were fitted using non-linear regression analysis (SigmaPlot v13.0, Systat, San Jose, CA, USA), with *R*^2^ ≥ 0.98, and *C*_t_ values for each environmental sample (beetle or pine stem tissue) were plotted against the appropriate standard curve to determine the amount of fungal DNA (ng) in each sample or the number of rDNA copies in each sample in the case of the 28S rDNA primers when compared with the gBlock control. A value of zero was used when no *C*_t_ was recorded or the recorded *C*_t_ value was below the limit of detection as determined by the standard curve.

### Sequence verification of rh-qPCR amplicons

Amplicons generated from fungal and beetle DNA using the 28S and Gc primers were verified via sequencing. The qPCR products were run out on a 1.5% agarose gel and extracted using a QiaQuick Gel Extraction Kit (Qiagen, Waltham, MA, USA). Purified amplicons were then ligated into the pGEM®-T vector (Promega Corporation, Madison, WI, USA) and transformed into DH5α *Escherichia coli* cells. Transformed cells were selected for on LB media supplemented with 100 μg ml^−1^ ampicillin (LB + Amp_(100)_) and 50 μl of 40 mg ml^−1^ X-gal (5-bromo-4-chloro-3-indolyl-d-galactoside) solution (Novagen, Millipore Sigma, Etobicoke, ON, Canada). Transformed single colony isolates were then grown shaking at 37 °C overnight in liquid LB + Amp_(100)_ media and plasmid DNA was purified using a QiaPrep Spin Miniprep Kit (Qiagen). Inserts were sequenced using the BigDye terminator sequencing system (Thermo Fisher Scientific, Waltham, MA, USA) with SP6 (5′-TATTTAGGTGACACTATAG-3′) and T7 (5′-TAATACGACTCACTATAGGG-3′) primers and sequenced on a 3730 DNA analyzer (Thermo Fisher Scientific).

### Statistical analyses

Fungal quantity values were log_2_ transformed (log_2_(*x* + 1) transformed in experiments with zero values) prior to statistical analyses. Levene’s test (median) was performed to test for homogeneity of variance before downstream analysis (*P* < 0.05). Significant differences in (i) effect of treatments on net photosynthetic rates and (ii) detection of fungal quantities and lesions lengths in seedling experiments were determined using ANOVA (*P* < 0.05). All statistical analysis was carried out using R v3.3.2 (R Development Core Team 2014).

## Results

### Histological detection of blue-stain fungi in lodgepole pine seedlings

**Figure 1.**
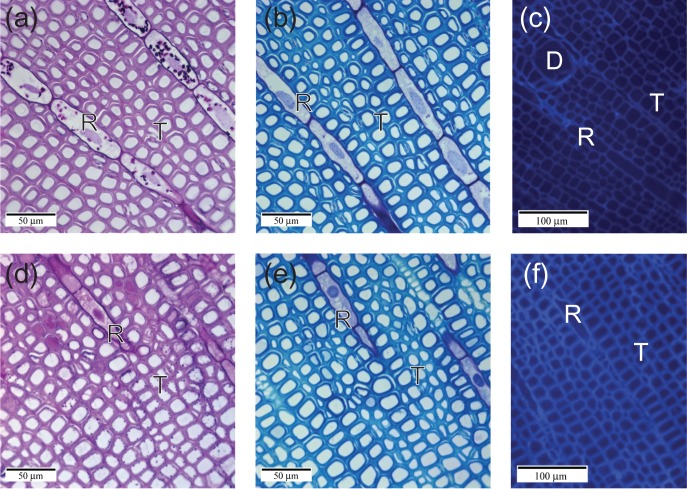
Histochemical examination of *G. clavigera*-inoculated lodgepole pine xylem using three classic stains for detection of fungal hyphae. (a–c) Uninoculated controls. (d–f) *Grosmannia clavigera*-inoculated stems collected at 42 dpi. Staining was with (a, d) PAS, (b, e) TBO, (c, f) Calcofluor White. No hyphae were detected using these three histochemical methods. R, ray; T, tracheid; D, resin duct.

To determine whether colonization of pine stems by *G. clavigera* resulted in sufficient fungal growth for visualization of mycelia—an indicator of fungal density in pine tissues—fixed sections of lodgepole pine stems collected at 42 dpi were stained with PAS, TBO and Calcofluor White (Figure 1). In agreement with our previous studies (Arango-Velez et al. 2014, 2016), while obvious structural changes were observed in *G. clavigera*-inoculated stems relative to the mock treatment, no fungal hyphae were visualized in any of the samples at any of the locations within the developing lesion (Figure 1).

### Detection of blue-stain fungi using rh-qPCR

**Figure 2.**
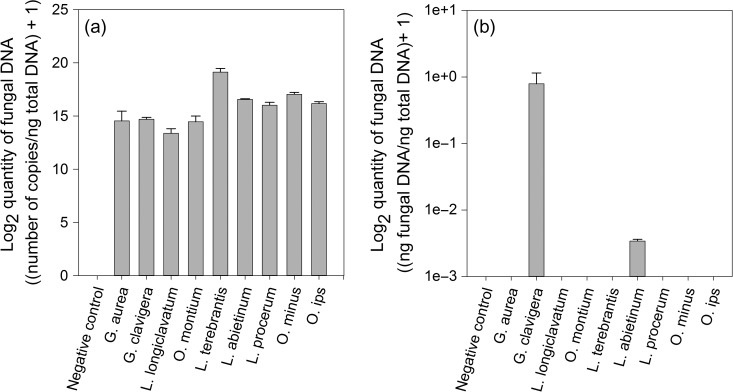
Detection of bark beetle-associated ophiostomoid fungi by rh-qPCR. DNA extracted from fungal cultures for nine species was analyzed by rh-qPCR with two primer sets: (a) 28S primers targeting an rDNA region conserved in ophiostomoid species examined in this study and (b) Gc primers targeting *β-tubulin* that were designed to distinguish *G. clavigera* from other MPB-associated ophiostomoid fungal species. Quantification was carried out using standard curves. For the 28S primer pair, the standard curve was prepared using a gBlock synthesized gene fragment, and values were calculated as the number of rDNA copies relative to the total DNA per reaction. For the Gc primer pair, the standard curve was constructed from *G. clavigera* DNA, and values calculated as ng *G. clavigera* DNA relative to the total DNA per reaction. Values are the mean ± SD for three strains each of species *G. aurea*, *G. clavigera*, *L. longiclavatum, O. montium* and *L. terebrantis* and one strain each of *L. abietinum*, *L. procerum*, *O. minus* and *O. ips*; three technical replicates were performed per strain.

Two sets of rh-qPCR primers were designed for detection of MPB-associated ophiostomoid fungi, based on the sequence data presented in Roe et al. (2010, 2011*b*), as outlined in Materials and methods. The first set were designed to amplify a conserved portion of the 28S region identified as shared by *G. clavigera*, *G. aurea*, *O. montium, L. longiclavatum* and *L. terebrantis* (Table 1). These 28S primers amplify a multi-copy region of the genome, which we hypothesized would increase sensitivity of detection within environmental samples such as pine stems or mountain pine beetles. The 28S primer set reliably detected nine different ophiostomoid species (Figure 2a), while no amplification was observed in the lodgepole pine control. Comparison of melt curves associated with biological samples to the gBlock (synthesized DNA sequence) positive control showed that the 28S primers resulted in a single, reliable product (see Figure S2 available as Supplementary Data at *Tree Physiology* Online). Sequencing of this product confirmed its identity as the targeted 28S rDNA region (data not shown).

*Grosmannia clavigera* is typically the most frequently isolated fungal symbiont species from MPB (Roe et al. 2011*a*), and is also considered the most pathogenic (Yamaoka et al. 1995, Bleiker and Six 2007, Plattner et al. 2008, Rice and Langor 2008). For these reasons, *G. clavigera* is often used as a proxy to simulate MPB attacks in ecological and physiological studies (Rice and Langor 2008, Arango-Velez et al. 2014, 2016), and is also the most often used species for investigations of MPB fungal associate–pine host interactions (Plattner et al. 2008). Since the rDNA qPCR primers used in Khadempour et al. (2012) for detection of MPB fungal associates in pine were unable to distinguish *G. clavigera* from *L. longiclavatum*, we also designed rh-qPCR primers against *G. clavigera β-tubulin* to discriminate *G. clavigera* from other blue-stain fungi associated with MPB, namely *G. aurea*, *O. montium*, *L. longiclavatum* and *L. terebrantis* (Table 1), while not amplifying off-target products in lodgepole pine. These Gc primers effectively detected *G. clavigera* but not *G. aurea*, *O. montium*,* L. longiclavatum* or *L. terebrantis*, and produced no amplification in the negative control lodgepole pine background (Figure 2b). We further tested the specificity of these Gc primers for *G. clavigera* by examining whether they amplified product from several ophiostomoid fungal species associated with other conifer bark beetles: *L. abietinum*, *L. procerum*, *O. minus* and *O. ips*. No amplification of *L. procerum*, *O. minus* or *O. ips* was observed (Figure 2b); however, limited amplification (at more than 100-fold lower levels) was detected for *L. abietinum*, a species associated with spruce beetle (*Dendroctonus rufipennis* (Kirby); Solheim and Safranyik 1997). Dissociation curves showed that, like the 28S primers, the Gc primers produced negligible off-target priming in the negative pine control (see Figure S2 available as Supplementary Data at *Tree Physiology* Online). These primers do produce a double peak in the positive fungal control (see Figure S2 available as Supplementary Data at *Tree Physiology* Online), even though only a single product corresponding to the expected amplicon was recovered by sequencing (data not shown). The identical double peak profile was also obtained in the inoculated pine samples, indicating no amplification of pine targets as suggested by the performance of the primers in the negative pine control.

For comparison, we also tested conventional qPCR primers against the *PCAS* target used by Khadempour et al. (2010). The conventional PCAS qPCR primers, which were slightly modified from those reported by Khadempour et al. (2010) to reduce primer dimers and improve amplification efficiency for detection of *G. clavigera* in pine samples, effectively detected *G. clavigera* cultured on media, but amplified an off-target locus when used to amplify *G. clavigera* from pine samples (see Figure S2 available as Supplementary Data at *Tree Physiology* Online).

### Quantification of *G. clavigera* in inoculated pine samples, and comparison with lesion length

**Figure 3.**
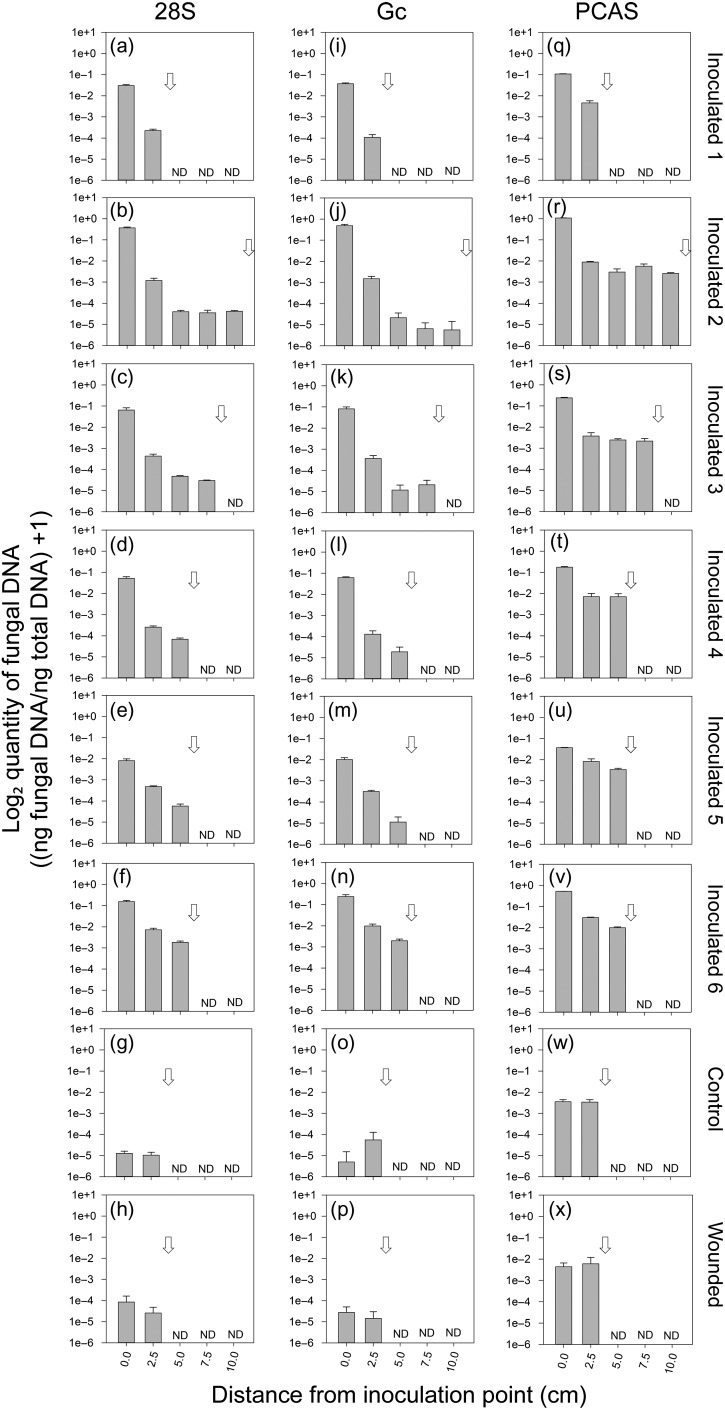
Quantification of *G. clavigera* along the vertical axis of lesions from mature lodgepole pine trees. Trunks of mature lodgepole pine were inoculated with *G. clavigera* (Inoculated 1–6; a–f, i–n and q–v), neither inoculated nor wounded (Control; g, o and w) or mock inoculated (Wounded; h, p and x), as described in Materials and methods. At 5 weeks after inoculation, lesions ranged in total length from 5 to 20 cm. A 1.25 cm leather punch was used to obtain xylem samples along the length of lesion, starting at the point of inoculation and extending down the length of the lesion at 2.5 cm increments to the edge of the lesion. Samples were analyzed with three separate primer pairs: (a–h) 28S rDNA ophiostomoid rh-primers (28S); (i–p) *G. clavigera* rh-primers (Gc) targeting the single copy *β-tubulin*; (q–x) *G. clavigera* PCAS conventional qPCR primers. Values were calculated using standard curves prepared using *G. clavigera* DNA, and are expressed relative to the total amount of DNA per reaction. Values are the log_2_ (ng fungal DNA ng^−1^ total DNA + 1) mean ± SD for each sample; three technical replicates were analyzed per sample. Control and Wounded trees were combined for analysis, *n* = 2 and *n* = 4, respectively. For inoculated samples, an arrow is used on each panel to indicate the extent of lesion development beyond the point of inoculation. ‘ND’ is used to indicate no data, i.e., distances from the inoculation point that were beyond the lesion limit for a particular replicate, and where no tissue was collected for analysis.

Having established that we could use the 28S primers to detect a suite of bark beetle-associated ophiostomoid fungi in the *Grosmannia* clade (sensu Zipfel et al. 2006), and that we could use the *β-tubulin* Gc primers to specifically distinguish *G. clavigera* from other *D. ponderosae*-associated ophiostomoid fungi, we examined whether we could use these primers to quantify *G. clavigera* in inoculated pine tissues, and if so, what the relationship would be between *G. clavigera* growth as determined by rh-qPCR quantification and lesion length. In the first experiment, we assayed xylem tissue harvested from within lesions of *G. clavigera*-inoculated mature lodgepole pine, using uninoculated unwounded and uninoculated wounded (mock) trees as controls. Total length of lesions from which tissues were sampled ranged from 5 to 20 cm (Figure 3; see Figure S3 available as Supplementary Data at *Tree Physiology* Online). We also used these samples to compare the efficacy of the 28S and Gc primers against the PCAS conventional qPCR primers (Khadempour et al. 2010). Fifty cycles were used to maximize the sensitivity of the rh-qPCR assay, but post hoc was determined not to be necessary to detect fungus in these pine samples. A standard curve for each primer set was used to quantify fungal DNA as well as to determine the linear dynamic range of the assay (see Figure S4 available as Supplementary Data at *Tree Physiology* Online). Samples with *C*_t_ values below the linear range of the standard curve were considered to be below detectable limits (<~0.0003ng ng^−1^ total DNA and ~0.005ng ng^−1^ total DNA for the 28S and Gc primers, respectively). The 28S and Gc rh-qPCR primers both amplified *G. clavigera* in the inoculated pine samples, while showing negligible amplification in the uninoculated control or uninoculated wounded pine samples (Figure 3). The PCAS conventional qPCR primers similarly amplified *G. clavigera* in the inoculated pine samples; however, as indicated in the previous section, they also showed considerable off-target amplification in both the uninoculated control and uninoculated wounded pine treatments (see Figure S2 available as Supplementary Data at *Tree Physiology* Online). Quantification using all three primer sets demonstrated that each of the six independent *G. clavigera*-inoculated trees showed the highest quantity of *G. clavigera* at the point of inoculation, with fungal quantity decreasing along the length of the lesion (Figure 3). The more fungal-specific 28S and Gc primers showed that *G. clavigera* quantities decreased to background levels well before the lesion margin in four replicates, and measurable quantities of *G. clavigera* in the sample closest to the lesion margin for two replicates. The PCAS primers amplified substantive product in all inoculated tissue samples along the lesion length, but the values obtained for most of these samples were comparable to the values obtained for the uninoculated control and uninoculated wounded samples.

**Figure 4.**
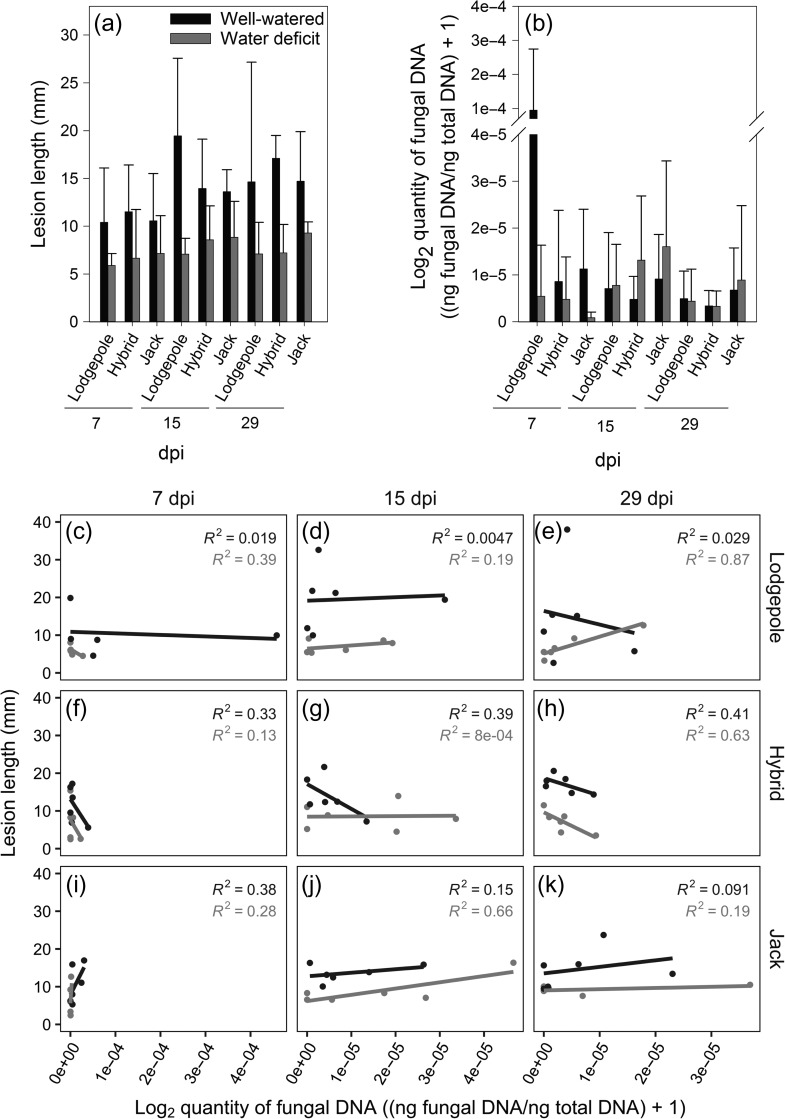
Relationship between lesion length and *G. clavigera* colonization as determined by rh-qPCR quantification in *G. clavigera*-inoculated lodgepole pine, jack pine and lodgepole × jack hybrid pine seedlings. Seedlings were subjected to well-watered or water-deficit conditions 4 weeks before inoculation and thereafter for the full duration of the experiment as described in Materials and methods. Lesion lengths and fungal quantification were measured for each seedling that was destructively harvested at three time points: 7, 15 and 29 dpi. In all panels, black denotes well-watered, while gray denotes water deficit. (a) Xylem lesion length determined by removing bark from inoculated stem sections; ±SD, *n* = 4–6. Lesion lengths are significantly affected by dpi (*P* = 0.0126) and water availability (*P* < 0.001); effect of species and the interaction terms are not significant (Table 3). (b) *G. clavigera* quantity as determined using 28S rDNA ophiostomoid rh-primers. Values were calculated using standard curves prepared using *G. clavigera* DNA (see Materials and methods), normalized to the total amount of DNA per reaction, and transformed using log_2_(*x* + 1); ± SD, *n* = 4–6. No factor was found to have a significant effect on *G. clavigera* quantity (Table 3). (c–k) Linear regressions of lesion length vs *G. clavigera* quantity data from (a) and (b), for each experimental condition. Linear regression analysis of the full dataset demonstrated no relationship between lesion length and *G. clavigera* quantity (see Figure S5 available as Supplementary Data at *Tree Physiology* Online).

In the second experiment, we assayed lesion-containing xylem tissue of *G. clavigera*-inoculated lodgepole pine, jack pine and lodgepole × jack pine hybrid second year seedlings subjected to well-watered or water-deficit conditions. The degree of imposed water deficit was sufficient to cause significant reductions in photosynthesis (see Figure S5 and Table S2 available as Supplementary Data at *Tree Physiology* Online), and was similar to that described in Arango-Velez et al. (2016). Lesion lengths (Figure 4; see Figure S3 available as Supplementary Data at *Tree Physiology* Online) were significantly different between water status treatments, and also between sampling dates (Table 3) but there were no significant differences observed between taxa, and none of the interactions was significant. In contrast to lesion length, there were no significant differences between quantities of *G. clavigera* detected in well-watered vs water-deficit trees, nor between species or sampling dates, largely because of the large amount of variance in fungal quantities (Figure 4; Table 3). Regression analysis of the full dataset revealed no relationship between lesion length and fungal quantity (*R*^2^ = 0.00041; see Figure S6 available as Supplementary Data at *Tree Physiology* Online). More detailed regression analyses within each water treatment/species/time point combination revealed that correlations between lesion length and fungal quantity generally improved within a single set of experimental conditions, with some experimental conditions exhibiting strong correlations (Figure 4). However, there was no evident pattern to the conditions—species, water status or days post-inoculated—that yielded higher vs lower correlations between lesion length and fungal quantity.

**Table 3.** Three-way ANOVA tables from statistical analysis of a full-factorial growth chamber study combining drought treatment with fungal inoculation in two pine species and their hybrid over three time points. Water treatment included two levels: well watered and water deficit. Experimental seedlings were in their second year of growth and were inoculated in two locations. A single asterisk indicates statistical significance at *P* < 0.05, while three asterisks indicate statistical significance at *P* < 0.001.

Experimental factors and interactions	Degrees of freedom	Three-way ANOVA: fungal content		Three-way ANOVA: lesion length	
		*F*-value	*P*-value	*F*-value	*P*-value
dpi	2	1.228	0.298	4.602	0.0126*
Water treatment	1	1.314	0.255	43.39	3.16E-09***
Pine taxon	2	1.129	0.328	0.012	0.9883
dpi × Water treatment	2	2.193	0.118	1.249	0.2918
dpi × Pine taxon	4	1.473	0.217	0.473	0.7551
Water treatment × Pine taxon	2	1.44	0.242	1.147	0.3222
dpi × Water treatment × Pine taxon	4	1.066	0.378	0.78	0.5411

### Species-specific detection and quantification of blue-stain fungi in situ in adult MBP

**Figure 5.**
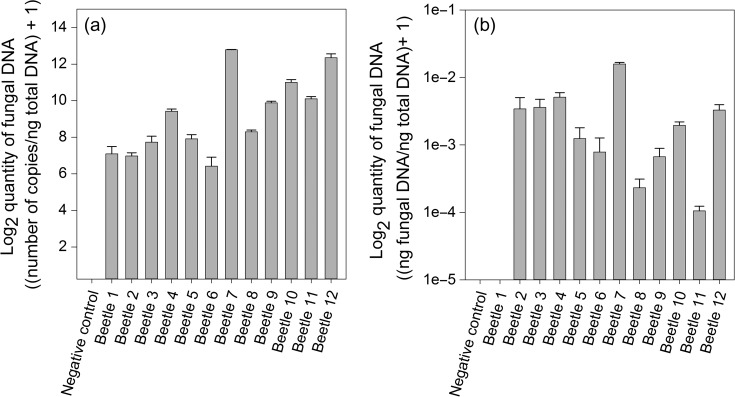
Quantification of blue-stain fungi in adult MPB by rh-qPCR. DNA extracted from individual adult beetles was analyzed by rh-qPCR with the (a) 28S primers targeting the rDNA region of ophiostomoid species examined in this study, or with the (b) Gc primers, targeting the *β-tubulin* region of *G. clavigera*. Values were calculated using standard curves prepared using (a) 28S gBlock or (b) *G. clavigera* DNA (see Materials and methods), normalized to the total amount of DNA per reaction. Values are the (a) log_2_ (number of copies ng^−1^ total DNA + 1) or (b) log_2_ (ng fungal DNA ng^−1^ total DNA + 1) mean ± SD for each beetle; three technical replicates were performed per beetle.

Given the utility of the 28S and Gc rh-qPCR primers to detect *G. clavigera* in *G. clavigera*-inoculated pine tissues, we also wanted to determine whether these rh-qPCR primers could be used to detect blue-stain fungi in adult mountain pine beetles from which DNA was non-selectively extracted. Based on the findings of Roe et al. (2011*a*, 2011*b*), we expected that these adult beetles would harbor multiple species of ophiostomoid fungi. Twelve adult beetles collected from MPB-attacked lodgepole pine in a heavily infested stand were ground, extracted and analyzed for the presence of ophiostomoid species using the 28S primer, and for *G. clavigera* using the Gc primer. Standard curves were used to calculate fungal quantity and determine the linear dynamic range. rh-qPCR using the 28S primers verified that all 12 beetles contained ophiostomoid fungal symbionts, as expected (Figure 5a). Analysis of the beetles using the Gc *β-tubulin* primers indicated that *G. clavigera* was detected in varying quantities in 11 of the 12 beetles (Figure 5b). Quantities of *G. clavigera* detected in Beetle 1 were below the threshold level of reliable detection as established via the standard curve linear dynamic range (<~0.0003 ng ng^−1^ total DNA and ~0.005 ng ng^−1^ total DNA for the 28S and Gc primers, respectively). As with the fungal amplicons above, sequencing of the rh-qPCR products verified that they were the targeted *β-tubulin* sequence (data not shown).

## Discussion

Forest pests and pathogens are an ever-present threat to the health and productivity of both natural and managed forests (Millar and Stephenson 2015, Wingfield et al. 2015). Weather patterns attributable to climate change, such as changes to moisture regimes (i.e., wetter periods or drought), milder winters and warmer summers, may exacerbate epidemics of native forest pests and pathogens (McDowell et al. 2011, Weed et al. 2013) while also facilitating range expansion of invasive pests and pathogens (Stenlid and Oliva 2016). Many species of bark beetles (Curculionidae: Scolytinae) and their fungal symbionts—including MPB and their associated ophiostomoid fungal pathogens—have exhibited massive outbreaks in recent years, in part because of climate change-related feedbacks (Raffa et al. 2008, Hulcr and Dunn 2011, McDowell et al. 2011). The ability to quantify a pathogen’s growth within its tree host is important for investigating host–pathogen interactions, effects of environmental cues on these interactions, and for identification of quantitative resistance to pathogen in tree populations (Sanzani et al. 2014). Advances in DNA diagnostic technologies such as PCR-based methods that improve sensitivity of detection and enable quantification thus have several applications in fundamental research and applied programmes.

Several methods have previously been described to detect and differentiate species of ophiostomoid fungi in MPB and pine samples, including microscopic observation (Ballard et al. 1984, Woo et al. 2005, Arango-Velez et al. 2014), fungal culturing from wood and/or insects (Solheim 1995, Adams and Six 2007, Roe et al. 2010, 2011*a*, 2011*b*), PCR of pure cultures and MPB DNA extracts (Khadempour et al. 2010), padlock PCR probes (Tsui et al. 2010) and qPCR of fungal-colonized pine tissues (Khadempour et al. 2012). These published methods each have limitations for robust in situ quantification of blue-stain fungal spore load or growth in environmental samples such as pine xylem or bark beetles. For example, conclusive detection of fungal hyphae by microscopy requires several months following fungal introduction into the tree, and also necessitates intricate sample preparation (Ballard et al. 1984, Woo et al. 2005, Arango-Velez et al. 2014, this study). Isolating blue-stain fungi from environmental samples such as wood or insects also requires substantial time and effort, is qualitative rather than quantitative, and may underestimate slow-growing or hard to culture species (Solheim 1995, Adams and Six 2007, Roe et al. 2011*b*). Similarly, conventional endpoint or padlock probe PCR—methods that were designed for detection and differentiation rather than quantification—provide only semi-quantitative estimates of abundance (Khadempour et al. 2010, Tsui et al. 2010). Additionally, endpoint PCR and variations such as nested PCR lack the sensitivity required to reproducibly detect the low amounts of ophiostomoid fungi present in pine stems at early time points following inoculation. Metagenomic approaches using next generation sequencing, such as has been recently used to identify the fungal community of the bark beetle *O. erosus* and the ambrosia beetle *X. saxesenii* (Malacrinò et al. 2017), and also the bacterial community of MPB (Adams et al. 2013), hold considerable promise for quantification of MPB-associated ophiostomoid fungal symbionts, but to our knowledge, this method has yet to be applied to the MPB fungal metagenome. Deep sequencing approaches also lack the precision and accuracy afforded by PCR-based techniques.

Because of the limitations posed by these methods, real-time qPCR has become the method of choice for detection of phytopathogenic fungi, bacteria and oomyctes in diverse plant tissues (reviewed in Sanzani et al. 2014) and other environmental samples such as soil (e.g., Almquist et al. 2016, Zhu et al. 2016). Recently, qPCR was used to quantify growth patterns of *Hymenoscyphus fraxineus* (T. Kowalski) Baral et al., the causative agent of ash dieback, in stems of *Fraxinus excelsior* trees, demonstrating the utility of this technology to understand pathogen invasion of the host tree (Matsiakh et al. 2016). Khadempour et al. (2012) developed a qPCR assay based on their endpoint PCR assay for quantitative detection of *G. clavigera*, *L. longiclavatum*, *O. montium* and *Ceratocystiopsis* sp.1, and used this assay to determine relative abundance of these fungal species in phloem samples lining and adjacent to MPB galleries. One of the limitations of the Khadempour et al. (2012) study was that the rDNA primers that effectively enabled detection of fungi in pine xylem did not distinguish *G. clavigera* and *L. longiclavatum*, unlike the *G. clavigera*-specific PCAS primers used by Khadempour et al. (2010) in their MPB fungal associate species diagnostic panel. The slightly modified PCAS primers that we used in this study amplified a single product in *G. clavigera*, but also amplified a non-target product in pine. As a result of this non-specificity, these primers led to consistent overestimation of *G. clavigera* fungal quantity in samples with low levels of fungal colonization, as demonstrated by the higher estimates of *G. clavigera* levels in the outer regions of lesions in the mature pine study.

These commonly encountered qPCR issues were circumvented by using the recently described RNase H2-dependent technology (Dobosy et al. 2011) to design primers targeting the same *G. clavigera PCAS* locus. In RNase H2-dependent PCR, which was originally developed for SNP variant detection, primers are designed with an RNA nucleotide near the 3′-end and blocked with a C3 spacer. The blocked primer prevents amplification by DNA polymerase (Dobosy et al. 2011). Thermostable *Pyroccocus abyssi* RNase H2 cleaves the primer 5′ to the RNA nucleotide, removing the blocking group and resulting in a 3′ OH, which allows DNA amplification to occur (Dobosy et al. 2011). RNase H2-mediated cleavage of the primer is optimal when the RNA nucleotide and the flanking DNA nucleotides are perfectly matched to the template DNA (Dobosy et al. 2011). As a result of RNase-H2 specificity, improperly annealed primers are not cleaved and remain blocked, preventing DNA amplification (Dobosy et al. 2011). This increases both specificity and sensitivity of the assay. To our knowledge, only one publication prior to this study has reported on the use of this technology, in which the authors used rh-qPCR to identify SNPs to determine if RNA editing was responsible for differences in DNA and RNA sequences for particular loci in *Chara* (Cahoon et al. 2017). Thus, the quantification application presented in this study is novel.

Once we established that rh-qPCR was more sensitive and target-specific than conventional qPCR, we designed sets of primers that reliably detected the presence of ophiostomoid species at very low levels in environmental samples as well as specifically detected *G. clavigera* within the MPB fungal microbiome (Roe et al. 2010, Roe et al. 2011*b*). We further demonstrated the utility of these primers to reliably distinguish fungal DNA in a background of MPB DNA or pine DNA. Use of standard curves enabled a more precise quantification beyond the relative quantification that had previously been reported (Khadempour et al. 2012). The primers that we have developed to *β-tubulin* were based on sequence data for more than 500 single spore isolates representative of different haplotypes of the five most commonly encountered MPB-associated ophiostomoid species compiled by Roe et al. (2010, 2011*b*). These *β-tubulin* rh-qPCR primers discriminate *G. clavigera* from the other four ophiostomoid fungi detected in MPB adults, larvae and proximal gallery tissue in MPB and proximal galleries by Roe et al. (2010, 2011*b*). Like *G. clavigera*, two of these fungal species, *L. longiclavatum* and *O. montium*, were frequently encountered, while *G. aurea* was infrequently encountered and *L. terebrantis* was rarely encountered, and only from galleries (Roe et al. 2010, 2011*b*). It is possible that *L. terebrantis* in these samples was a result of co-colonization of trees by secondary bark beetles such as *Ips* or ambrosia beetles, as has been suggested by Lee et al. (2006). These primers exhibited limited detection of *L. abietinum*, but as this ophiostomoid species is associated with spruce bark beetle (Solheim and Safranyik 1997), there is little probability that this species would be found in the MPB fungal microbiome or otherwise incidentally associated with the pine host.

Because pathogen DNA generally represents a very small proportion of the overall DNA extracted from the host tissue, increased sensitivity—effectively increasing the crossing threshold (*C*_t_) at which reliable quantification can occur—is important (Grosdidier et al. 2017). We thus sought to improve further the sensitivity of the rh-qPCR assay by (i) targeting a multi-copy locus and (ii) increasing the number of PCR amplification cycles. The multi-copy nature of the 28S rDNA made it a good candidate for ascertaining whether targeting a multi-copy fungal locus would increase sensitivity of fungal detection in MPB or pine tissues. This 28S region and neighboring regions were particularly attractive because these regions and the larger ITS region are commonly used in fungal species diagnostics (reviewed in Tedersoo and Lindahl 2016), and had previously been used for detection of ophiostomoid MPB symbionts (Khadempour et al. 2010, 2012, Roe et al. 2010, 2011*b*, Tsui et al. 2010). Affirming our expectations, targeting the multi-copy 28S resulted in greater sensitivity than targeting the single-copy *β-tubulin*, as evidenced by the ability to significantly measure fungal DNA in a greater number of tested pine samples. However, this region of the genome is GC-rich and relatively conserved, precluding the possibility of designing effective species-discriminating primers. A measureable degree of non-target signal was obtained with the 28S primers at ~45 cycles, possibly due to amplification of non-ophiostomoid fungal endophyte DNA present in the control samples. This small but measurable increase in off-target amplification after 45 cycles using the 28S primers was offset by the ability to significantly detect *G. clavigera* at lower abundance than with the β-tubulin primers. Accordingly, either or both primer sets can be used, according to whether specificity or sensitivity is most germane to the objectives of the analyses to be carried out.

Lesion length is commonly used to assess tree defenses against bark beetle fungal associates, including ophiostomoid fungi vectored by MPB (e.g., Wallin and Raffa 2001, Masuya et al. 2003, Rice et al. 2007, Plattner et al. 2008, Lu et al. 2010, Addison et al. 2013, Goodsman et al. 2013, Arango-Velez et al. 2014). We used rh-qPCR to assess the relationship between lesion length and (i) pattern of *G. clavigera* growth within this lesion and (ii) *G. clavigera* quantity within the stem volume containing the lesion. Our ability to detect *G. clavigera* DNA in the lesion at the point of inoculation and more distally from the point of inoculation was more accurate and precise than we could obtain using conventional qPCR with primers somewhat modified from those of Khadempour et al. (2010), demonstrating the utility of this method to rigorously examine this question. Of the six different lesions from independent *G. clavigera*-inoculated mature trees that we surveyed, most showed little fungal quantity in the distal portion of the lesion, particularly those trees that showed longer lesions. However, at least two of these lesions showed levels of *G. clavigera* in excess of background (i.e., values greater than the residual levels obtained in the two control treatments) near the end of the lesion. Thus, we cannot make the inference that lesion length reflects the extent of fungal growth, as detectable by rh-qPCR. Because measurable fungal quantities were present near the lesion margin in a proportion of the lesions that were analyzed, this suggests that further analyses are warranted to determine if MPB-associated ophiostomoid fungal colonization can extend beyond the margin of a lesion.

The level of water limitation imposed in this study was sufficient to significantly impact photosynthetic capacity. In previous studies, decreased photosynthetic capacity of this magnitude in lodgepole pine, jack pine and lodgepole × jack pine hybrids has been associated with attenuated induced defenses to *G. clavigera* such as reduced induction of specific monoterpenes, reduced traumatic resin duct production and reduced upregulation of defense-associated genes such as chitinases (Arango-Velez et al. 2014, 2016). Our analyses with *G. clavigera*-inoculated seedlings indicated that while factors such as water deficit and time following inoculation significantly affected lesion development, these factors had no significant effect on fungal quantity. Across all treatments, there was no correlation between lesion length and fungal colonization, although a few specific treatment combinations did yield high correlations between lesion length and fungal quantity. The varying degrees of correlation between lesion length and *G. clavigera* quantity further suggest that the dynamics of lesion development and *G. clavigera* growth are differentially affected by factors such as host taxa, host water status and time following inoculation. This lack of a clear relationship may reflect that lesion length does not provide a measure of the extent to which ophiostomoid fungal colonization penetrates radially into the sapwood, which ultimately triggers tracheid cavitation via tyloses (see Arango-Velez et al. 2016). Based on this, we hypothesize that this observed variation in lesion length—*G. clavigera* quantity correlation reflects more radial growth of *G. clavigera* in some treatment conditions than others.

In Arango-Velez et al. (2016), we proposed that the significant decrease observed in *G. clavigera*-inoculated lodgepole pine hydraulic conductivity but not in *G. clavigera*-inoculated jack pine was due to increased early colonization by *G. clavigera* in lodgepole pine, resulting in a greater degree of tyloses. We were unable to provide experimental support for this theory with the rh-qPCR analyses presented in this study: the observed increased levels of *G. clavigera* in lodgepole pine seedlings at 7 dpi compared to jack pine were not significant, largely owing to the high standard deviation between biological replicates obtained for the fungal quantification study. A relatively high variance was also observed in lesion length between these same biological replicates. This high degree of variation between biological replicates in both the seedling and the mature tree studies may have arisen from having used provenance rather than clonal plant genetic material for these experiments, as is common practice for investigations with conifer species. We hypothesize that the observed variation reflects differing levels of genetic susceptibility to *G. clavigera* between these biological replicates that is not evident from measurements of lesion length. Ongoing experiments in our laboratory are testing this hypothesis.

Together, these analyses demonstrate that rh-qPCR is an appropriate method to investigate questions such as environmental influences on tree defense against MPB-associated ophiostomoid fungi, differential virulence between MPB-associated ophiostomoid fungal genotypes within a species or between species (e.g., Rice et al. 2007) and relative resistance of host genotypes within a species or between species. The latter approach mirrors the quantification of fungal growth by qPCR that is commonly used to assess relative resistance of crop species against pathogens, particularly foliar pathogens (e.g., Llorente et al. 2010, Knüfer et al. 2017).

### Conclusion

RNase H2-dependent qPCR is a recently developed modification of qPCR (Dobosy et al. 2011) that shows greater sensitivity and specificity than standard PCR or qPCR methods for detection, differentiation and quantification of microbial DNA within environmental samples such as insect or plant tissues. Here, we have demonstrated the utility of rh-qPCR to quantify ophiostomoid fungi in situ from MPB and pine DNA extracts, with a focus on the most prevalent and pathogenic MPB fungal associate, *G. clavigera*. This is a novel application of rh-qPCR, which was originally designed for SNP detection of rare variants (Dobosy et al. 2011). We used the technology to demonstrate that lesion length is not an accurate predictor of the extent of *G. clavigera* growth along the lesion axis, nor total fungal growth within the infected area, and that this relationship may be affected by variables such as duration of host–pathogen interaction, host genotype and host vigor. These findings underscore the need to explore the relationship between fungal colonization of the host tree and resulting lesion development in greater detail using sensitive and specific molecular approaches such as the rh-qPCR that we describe here. To be able to explore this relationship in naturally MPB-infested trees—which typically carry more than one MPB-associated ophiostomoid species—will require a panel of rh-qPCR primers that effectively detect and discriminate multiple ophiostomoid members of the *D. ponderosae* microbiome. This research is ongoing in our laboratory.

Beyond the application reported here, rh-qPCR technology can be applied to any area of research where discrimination and/or quantification of low-abundance DNA or RNA is desired. rh-qPCR is a straightforward and cost-effective way to improve specificity and sensitivity of quantification by real-time PCR compared with use of sequence-specific quenched fluorescent probes (e.g., TaqMan), thereby making rh-qPCR attractive for routine or high-throughput analyses or in scenarios in which diagnosis and quantification of many different pathogens or other microbes is necessary.

## Supplementary Material

Supplementary Data

Supplementary Data
