# Supplementary material for: A novel application of RNase H2-dependent quantitative PCR for detection and quantification of Grosmannia clavigera, a mountain pine beetle fungal symbiont, in environmental samples
Source: Tree Physiol. 2018 Jan 10;38(3):485–501. doi: 10.1093/treephys/tpx147 (PMC5982843; doi:10.1093/treephys/tpx147)

Table S1. NCBI accession numbers for 28S rDNA and β-tubulin loci that were sequenced by Roe et al. (2011) to establish the identity of each of the isolates used for the present study.

Species	UAMH ID	Unique ID from Roe et al. (2011)	NCBI Accession Number 28S rDNA	β-tubulin
G. clavigera	11139	M001-02-03-05-UC17DL22	GU370273	GU370187
G. clavigera	11143	M002-06-01-03-UM01G12	GU370274	GU370188
G. clavigera	11147	M002-12-03-03-UC10G11	GU370298	GU370212
G. aurea	10965	M001-03-03-06-UC03DL06	GU370267	GU370181
G. aurea	10969	M002-01-03-02-UC12DL18	GU370293	GU370207
G. aurea	10970	M002-06-01-08-UL05DL39	GU370260	GU370174
O. montium	11037	M001-02-01-01-UM02DL02 SS196	HQ413587	HQ413435
O. montium	11038	M001-02-01-07-UM34G38 SS186	HQ413584	HQ413432
O. montium	11043	M001-13-03-03-UM04G04 SS315	HQ413608	HQ413456
L. longiclavatum	11014	M002-12-03-03-UC10G11	GU370282	GU370196
L. longiclavatum	11017	M002-03-01-17-UC47DA13G63	GU370279	GU370193
L. longiclavatum	11019	M002-06-05-14-UL03DL74	GU370299	GU370213

Figure S1. gBlock sequence containing the conserved partial 28S rDNA region of ophiostomatoid fungi. Regions of 28S primer binding are underlined. Bolded letter within this underlined sequence denotes the single point at which nucleotide variation exists among *G. clavigera*, *G. aurea*, *L. longiclavatum* and *O. montium* within the specified region.

5’-gttccttggaacaggacgccatagagggtgagagccccgtacgggcggacgcctagcct**c**tgtgaagctccttc
gacgagtcgagtagtttgggaatgctgctcaaaatgggaggtaaatttcttctaaagctaaataccggccagagaccg
atagcgcacaagtagagtgatcgaaagatgaaaagcactttgaaaagagggttaaaaagtacgtgaaattgttgaaag
ggaagcgcctgtgaccagacttg-3’

Deleted: Supporting Information¶
¶

Figure S2. Comparison of *G. clavigera* detection using regular primers and rh-PCR technology at three different loci. Three separate primer sets were used to detect *G. clavigera* in DNA extracted from a *G. clavigera* culture (row 1), uninoculated mature lodgepole pine xylem (row 2), and *G. clavigera*-inoculated mature lodgepole pine xylem sampled within the resulting lesion at the point of inoculation (row 3) or 2.5 cm away from the site of inoculation (row 4). Column 1: 28S ophiostomoid *Grosmannia* clade rh-qPCR primers targeting the multi-copy *28S* rDNA region. Column 2: *G. clavigera* selective rh-qPCR primers targeting the single copy *β-tubulin*. Column 3: PCAS conventional qPCR primers targeting the *peroxisomal-coenzyme A synthetase* (*PCAS*) gene (Genbank HQ633743) used by Khadempour et al. (2010). Two to three technical replicates are shown per dissociation curve. The 28S primers produce a single, reproducible peak in *G. clavigera*-containing samples, but no off-target amplification in the uninoculated pine sample. Sequencing of this single amplicon verified the expected product. The *G. clavigera* selective primers similarly show amplification in *G. clavigera*-containing samples, but negligible to no signal in the uninoculated pine negative control. Two peaks are consistently obtained for this primer set, but only a single product with the expected sequence was obtained by sequencing the resulting amplicon. The PCAS conventional qPCR primers yield different products in the *G. clavigera* positive control and the uninoculated pine negative control. Both products are clearly visible in the *G. clavigera*-inoculated pine samples.

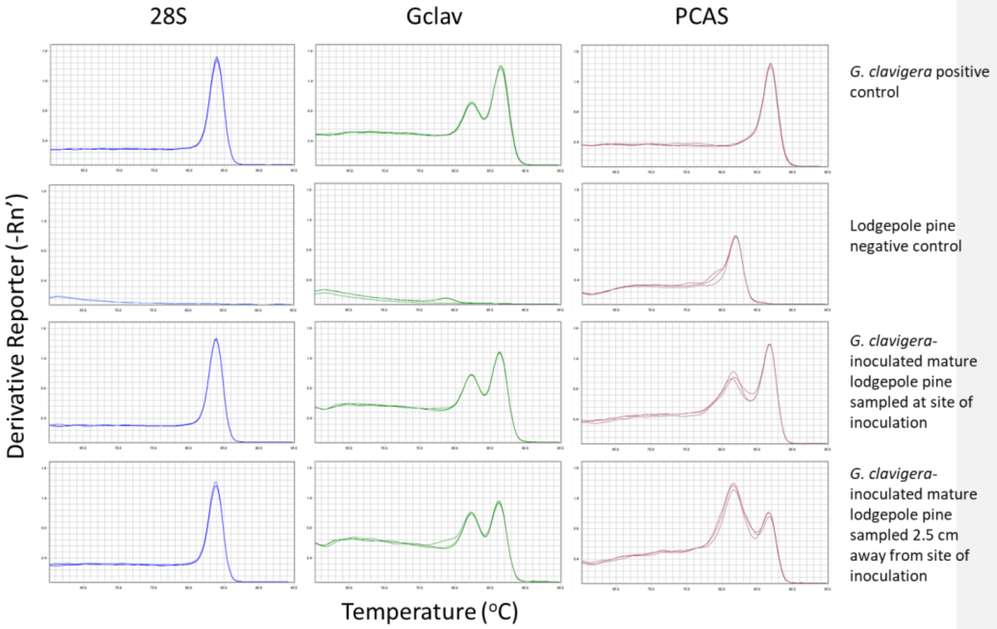

Figure S3. Wounded uninoculated and *G. clavigera*-inoculated pines showing examples of lesion development, corresponding to data in Figures 3 and 4 in the main text. (a) Wounded uninoculated mature lodgepole pine five weeks following mock inoculation; (b) *G. clavigera*-inoculated mature lodgepole pine five weeks following inoculation; (c) *G. clavigera*-inoculated 2-year-old lodgepole pine seedlings, 29 dpi.

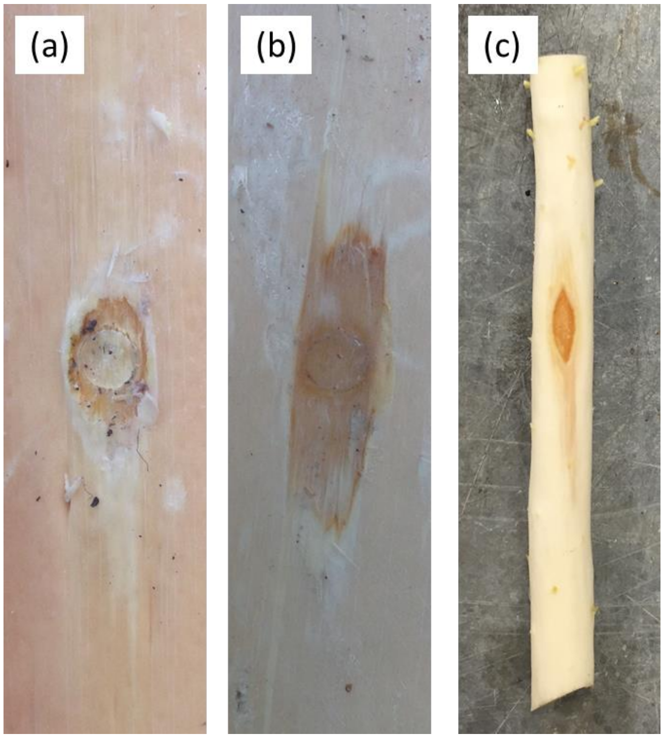

Figure S4. Quantification of ophiostomatoid DNA in *G. clavigera* inoculated lodgepole pine seedlings using regression analysis. Standard curves were produced using (a) dilutions of the gBlock double-stranded synthesized gene fragment containing the conserved partial 28S rDNA region for detection using the 28S rh-qPCR assay; (b) DNA isolated from cultured *G. clavigera* using the 28S rh-qPCR assay; (c) DNA isolated from cultured *G. clavigera* using the Gc rh-qPCR assay ;(d) DNA isolated from cultured *G. clavigera* using the conventional PCAS qPCR assay. Non-linear regression analysis was used to fit standard curves. Additional points (not shown) both at higher and lower values than the points illustrated here were originally assayed to establish the linear dynamic range for each standard curve. Values represent mean ± SD, n=3.

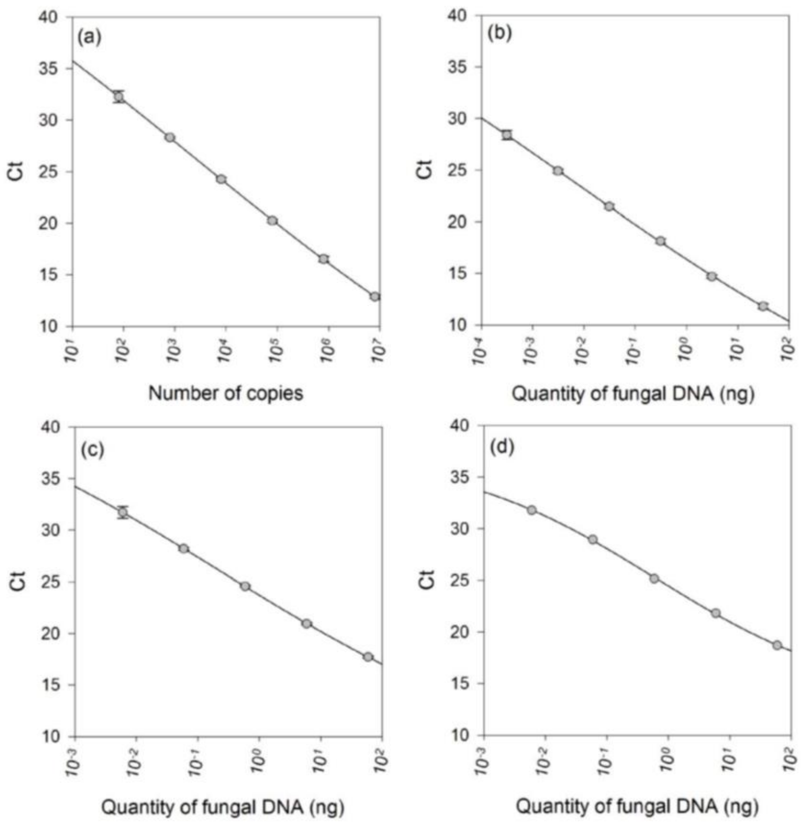

Figure S5. Effect of water deficit on the photosynthetic capacity (A_{max}) of *G. clavigera*-inoculated lodgepole pine, jack pine and lodgepole x jack hybrid pine seedling. Seedlings were subjected to well-watered or water deficit conditions for four weeks prior to inoculation, and thereafter for the full duration of the experiment as described in Materials and Methods. A_{max} was measured at 1 day post inoculation (dpi), 6 dpi, 14 dpi and 28 dpi using a Li-6400 (LI-COR Biosciences, Lincoln, NE, USA). Photosynthetic capacity was significantly affected by water deficit, as well as dpi and species; no interactions of this factors were significant (Table S1). (a) Boxplots of photosynthetic capacity over time with species individually plotted, n=11-12.(b) Boxplots of photosynthetic capacity over time with species grouped together, n=35-36.

Formatted: Font: Not Bold

Formatted: Font: Not Bold, Subscript

Formatted: Font: Not Bold

Formatted: Font: Italic

Formatted: Font: Not Bold

Formatted: Font: Not Bold

Formatted: Font: Not Bold

Formatted: Font: Not Bold

Formatted: Subscript

Formatted: Font: Not Bold

Formatted: Font: Not Bold

Formatted: Font: Not Bold

Formatted: Font: Not Bold

Formatted: Font: Not Bold

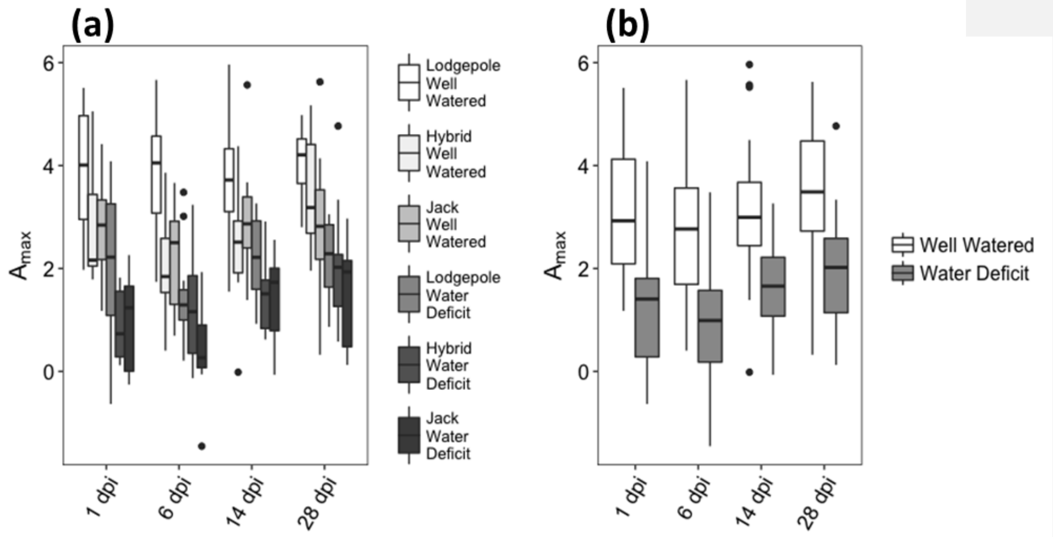

Table S2: ANOVA of the full factorial experiment depicted in Figure S5 comparing effect of water availability, time following inoculation and taxa on photosynthetic capacity (A_{max}) of *G. clavigera*-inoculated lodgepole pine, jack pine and lodgepole x jack pine hybrids. Water treatment included two levels: well-watered and water deficit. Photosynthesis was measured using a LI-COR Li-6400 at 4 time points: 1 day post-inoculation (dpi), 6 dpi, 14 dpi and 28 dpi. Two-year-old seedlings were inoculated with *G. clavigera* as described in Materials and Methods at two locations on the main stem 4 weeks after initiation of water treatments.

**Experimental factors and interactions**	**Degrees of freedom**	**Three-way ANOVA: Photosynthesis**	
		F-value	P-value
DPI	3	7.110	0.000131***
Water Treatment	1	165.968	<2e-16***
Pine taxa	2	27.303	1.72e-11***
DPI: Water treatment	3	0.603	0.613786
DPI: Taxa	6	1.069	0.381408
Water treatment: Taxa	2	2.126	0.121386
DPI: Water treatment: Taxa	6	0.689	0.658768

***statistically significant at P<0.001

Formatted: Font: Bold

Formatted: Subscript

Formatted: Font: Italic

Formatted: Font: Italic

Figure S6. Regression of xylem lesion length versus *G. clavigera* quantification in *G. clavigera*-inoculated lodgepole pine, jack pine and lodgepole x jack hybrid pine seedlings subjected to either well-watered or water deficit conditions. Water treatments were initiated four weeks before inoculations were conducted, and continued for the duration of the experiment. Seedlings were inoculated in two locations on the first year main stem by puncturing bark with a blunt tipped needle and pipetting 1 µL of *G. clavigera* spore suspension onto exposed xylem. Lesion lengths and fungal quantification were determined as paired measurements on seedlings destructively harvested at three time points: 7, 15 and 29 dpi. n=4-6 for each time point, water treatment and species combination. Fungal quantity was determined using 28S rDNA ophiostomoid rh-primers (28S) with 40 cycles of qPCR. Values were calculated using standard curves prepared using *G. clavigera* DNA, are expressed relative to the total amount of DNA per reaction, and are transformed using $log_2(x+1)$. Linear regression was conducted in R.

Deleted: 5

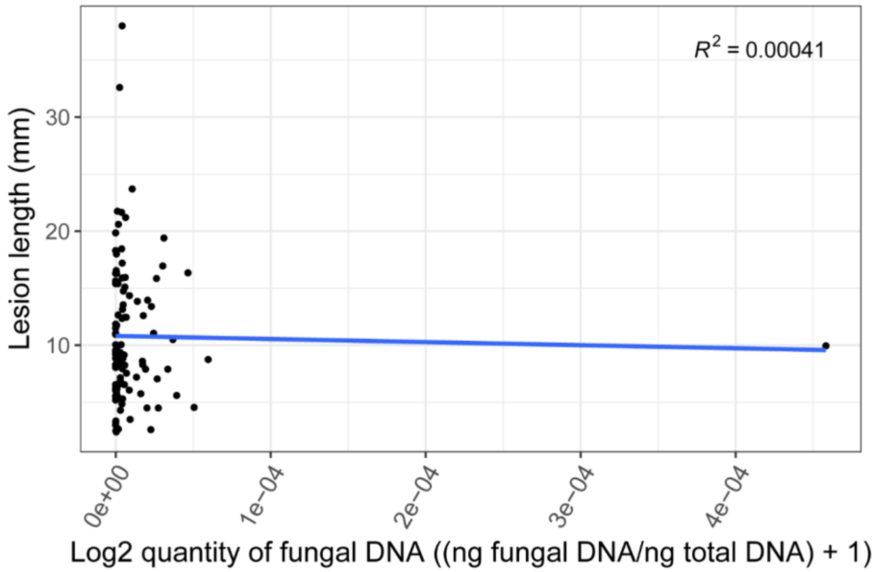

Supplement: Supplementary Data [file supplementary_data_combined_markup.pdf]
